# Bibliographical Notices

**Published:** 1848-10

**Authors:** 


### EDITORIAL DEPARTMENT.

### BIBLIOGRAPHICAL NOTICES.

*On the Causes and Treatment of Abortion and Sterility: being the result of an extended practical inquiry into the Physiological and Morbid conditions of the Uterus, with reference especially to Leucorrhœal Affections, and the diseases of Menstruation.* By James Whitehead, F. R. C. S., Surgeon to the Manchester and Sæford Lying-in Hospital. Philadelphia, Lea & Blanchard, 1848. 8 vo., pp. 368.

One hundred and fifty pages of this work are devoted to Menstruation, considered physiologically and pathologically. The signs of Pregnancy are next considered, occupying forty-six pages. Next, follows the subject of abortion, statistics with reference to various points, and full consideration of the causes. Lastly, *Sterility, its causes and treatment.* Without containing much that is new, the author, from the extensive opportunities of investigation which he has enjoyed, and of which he appears to have availed himself industriously, has contributed a body of facts, important in their bearing upon various interesting topics, and which go to establish certain points with greater precision than previous investigations had done.

The concluding chapter on sterility, is, practically, the most valuable portion of the work. As his remarks on the cause of sterility which, in his opinion, is the most frequent, do not cover an extended space, we transfer them to our columns:—

"Chronic endo-uteritis, or what may be called irritable uterus, is, in fact, one of the most frequent causes of sterility. The disease generally sets in soon after marriage, in cases where it is not attributable to specific causes; and in some rare instances, there are evidences of its having existed previously. The earliest symptoms are, a sense of tension and soreness of the abdomen, aching of the loins, pain of the hypogastrium and thighs, pallor of the countenance, languor, and general *malaise.* A slight leucorrhœal discharge manifests itself; this, at first, is clear and colorless, but soon acquires a yellowish or brownish tinge, and is not unfrequently offensive. The menstrual functions are performed with suffering; the discharge appears in unusual abundance, being sometimes clotted; and the peculiar secretion characteristic of internal inflammatory action, occupies the whole interval between one menstrual period and another. The affection is accompanied with rigors and remittant fever; and there is a great tendency to peritoneal and enteritic disturbance. If the uterus be submitted to examination, its body, especially at the posterior aspect, will generally be found enlarged and painful to the touch, the cervix is unusually tense, sometimes erysipelatous, the labia are more or less thickened, and present a vivid red, irritable-looking ring of inflammation surrounding the orificium uteri, whence issues a quantity of ichorous or sanious fluid, emmitting a peculiar odor. For the most part, this fluid product differs materially in its sensible properties both from pus and the normal uterine mucus; the two latter are essentially alkaline; whereas the former exhibits an acid reaction, which property, on some occasions, upon subsequent admixture with the vaginal mucus, becomes sufficiently intense to inflame and excoriate the external parts with which, in escaping, the product is necessarily brought into contact.

The prevention of pregnancy under these circumstances, may be occasioned in three ways: *in the first place*, the inflammatory action going on within the uterus, and which is liable to be aggravated under states of venereal excitement, may prevent the formation of the *membrana decidua*, and the ovum, even although impregnated, is necessarily thrown off without any manifestation of its existence in the fertilized state; *secondly*, the diseased condition of the lining membrane of the uterus may be extended to the Fallopian canals, obliterating for the time their internal orifices, so as to oppose an insurmountable obstacle to the admission of the spermatic fluid within them, and thus to render the fertilizing effort abortive; *thirdly*, the nature of the secretion furnished by the internal surface of the uterus or of the vagina, under certain states of disease, may be inimical to the active existence of the spermatozoa, occasioning their destruction before they arrive at the extricated ovule. The conditions under which the two first propositions are capable of being verified, have been sufficiently dwelt upon already; in referring to the latter, a few brief remarks may not be inappropriate in this place.

The spermatozoa (or spermatozoides, as they have recently been termed by several eminent physiologists, who seem inclined to question their existence as independent animalcules,) are alone contained in the spermatic fluid of the male, of which they constitute the essential or fecundating property. By their non-existence therefore in this secretion, the fluid is deprived of its fertilizing influence; this may at least be reasonably inferred, as observed by Dr. Carpenter, from several circumstances, such as their absence or imperfect developement in hybrid animals, which are nearly or entirely sterile: and the fact that fecundation essentially consists in the direct communication of one of them with a certain point in the ovum. Various opinions are entertained respecting the precise point at which the two elements of germination are brought into contact; some having stated that the spermatic animalcule has been found to have traversed the whole length of the Fallopian canal, while the ovule was still contained within the cavity of the corpus fimbriatum; in other instances, their communication has been witnessed in the course of the tube, sometimes nearer its inner extremity, or even after the ovule has been deposited in the cavity of the uterus. The last-named situation, it has been contended, is that at which impregnation is probably always effected under normal circumstances; but the occasional occurrence of extra-uterine fœtation proves that fertilization may take place at any point between the ovarium and uterus.

The spermatic animalcules are capable of living for a length of time in an isolated condition, their existence being prolonged or otherwise according to the nature of the media with which they are brought into contact after emission. Thus, in healthy vaginal mucus collected from females after copulation, they are seen to move actively for several days: while the product of the same organ, or that of the uterus, in certain states of disease, will cause them to perish instantly. This fact has been demonstrated by M. Donné, who, in his experiments, employed the mucus of both these organs, separately and conjunctly, in their respective states of health and disease. At page 291 of his work (*Cours de Microscopie,*) M. Donné observes: "As might be expected, the Zoospermes (Spermatozoa) live perfectly in the mucus secreted by the vagina in its normal state." "But," he prcceeds in the following page, "the vaginal mucus becomes so acid in some circumstances, as in cases of congestion, irritation, or inflammation of this organ, that the Zoospermes appear to perish in a few seconds after being brought into contact with it. The same effect was also produced by the vaginal mucus secreted during pregnancy; this fluid exhibiting its acid properties in a much higher degree of intensity in the gravid than in the unoccupied state of the uterus." The latter fact I have long been familiar with, but have not had an opportunity of witnessing the effect of this product upon the spermatic fluid under the microscope. M. Donné says: "What seems to me remarkable is, that the vaginal mucus of pregnant women, has generally appeared to be inimical to the existence of the Zoosperme; and, in fact, the state of congestion which exists in these parts during pregnancy, is that under which the acidity of the vaginal mucus ordinarily becomes very decided." He appears to be unaware of the fact, however, that the secretion of the vagina and that of the uterus, in their healthy condition, are essentially different in their chemical properties; hence the conclusion arrived at by him respecting the cause of the phenomenon in question;—a conclusion which I have reason to believe to be erroneous. It is highly probable that the same proportion of the acid component is eliminated by the secreting apparatus of the vagina, both during pregnancy and at other times; the remarkable difference noticed in the two states, being determined by the presence or absence of a powerful modifying agent—the uterine mucus, the formation of which is necessarily suspended during pregnancy. I have before endeavored to show on testimony of experiment, that uterine mucus in its healthy state, exhibits an alkaline reaction, while that of the vaginal secretion under like circumstances, is always acid; and although in the product resulting from their combination, the latter generally appears as the predominant property—constituting in fact the distinguishing characteristic of this fluid; nevertheless the tendency of their union is constantly towards neutralization; consequently, the highly acidified state of this product can only result either from withdrawal of the uterine mucus, as during pregnancy, or from change of its properties by disease. We have here then within easy accomplishment—if M. Donné's statement respecting the destructive nature of the changed secretion should hereafter be found correct and of general application—a means whereby satisfactorily to elucidate the pathology of sterility, in some women at least, as well as several other important phenomena connected with this branch of physiology, which have heretofore been considered inexplicable upon scientific principles. But to proceed.

In reference to the effect produced upon the vitality of the seminal fluid by contact with *uterine mucus,* M. Donne remarks at page 294: "Generally speaking, the Zoospermes brought into contact with uterine mucus, do not appear to experience any deleterious influence, even when this product has lost its state of purity, and become more or less purulent." "But," he proceeds, "certain kinds of uterine mucus, kill the animalcules with the greatest rapidity; thus, while they continue lively and active for several hours in the secretion taken from one woman, they appear to perish on the instant, and to lose entirely the power of motion, in that taken at the same time from another person; the experiment being performed under like circumstances in both cases." This author considers that the deleterious action exercised by certain mucous products of the uterus upon spermatozoa, is to be attributed to the excess of alkali which the secretion appears to contain in some women, or in some states of disease. I am inclined, however, to question the validity of this statement; having grounds for believing that the condition most frequently and most powerfully effective in preventing fecundation, is that under which the secretion becomes endowed with a property precisely the opposite of that which he assumes to be peculiarly deleterious.

I look upon the statements of the author just quoted, in addition to results obtained in the course of my own observations already noticed in several parts of this volume, together with others to be presently adduced, as constituting a body of facts of no mean importance in the study of physiology and morbid changes of the uterus. It should be borne in mind, to reiterate the circumstance, that uterine mucus, both in its healthy state, as well as under certain morbid conditions, exhibits an alkaline reaction. This I have ascertained by experiment more than a hundred times repeated. Its tendency is to modify the opposite quality possessed by the mucus of the vagina. But in chronic endo-uteritis, and perhaps in other forms of disease also, its nature is entirely different, and instead of the salutary change which, under normal circumstances, it operates upon the product with which it becomes incorporated, an effect the opposite of this is produced. If vaginal mucus therefore, in the highly acidified state in which it is found when deprived of its other component, be proved to be inimical to the existence of the spermatozoa: it is not unreasonable to infer that the same deleterious property will also be possessed by it when the uterine secretion is withheld from other causes; and its nature will be still more energetically destructive in those cases wherein both are possessed of similar properties."

The work concludes with reports of several cases illustrative of the efficacy of treatment in conformity with the pathological views enunciated in the foregoing remarks. As the consideration of the subject is not complete without an account of the local therapeutical measures which the author recommends, we make the following extract from another portion of the work relating to that point:—

"The treatment consists in the application of leeches to the *hypogastrium*, or over the upper part of the *sacrum;* and in the exhibition of remedies similar to those employed in CASE XXI. In addition, when all febrile excitement has become completely subdued, a more decidedly tonic plan will probably be indicated; and considerable benefit may be derived from injecting within the *uterus*, a weak solution of nitrate of silver in combination with *extractum conii*;* or by the introduction of an ointment of the same material.† The latter form of the remedy is applied by means of a small piece of lint fixed upon the end of a long probe, and fastened to the handle of the instrument with a thread, with a view of securing its safe withdrawal. Not only does no dirturbance or discomfort of any kind ensue upon the employment of these local measures; but, on the contrary, a beneficial change is often evident after the first application, and I have witnessed the suspension of pain to be instantaneous. The operation of injecting is done with the aid of the speculum, by means of a long syringe similar to Clarke's female syringe, having a nearly straight tube, with but one hole at its extremity. If carefully managed, from one to two drachms of the remedy, (which may be composed of different materials, varied in form and strength according to circumstances)—will pass within the organ at each operation, most of which returns almost immediately. The temperature of the fluid, previous to being used, should be raised to about ninety degrees, Fahr.

---

*Females and their Diseases; a series of Letters to his Class.* By CHARLES D. MEIGS, M. D., Prof. of Midwifery, and the Diseases of Women and Children, in the Jefferson Medical College at Philadelphia, &c., &c., &c. Philadelphia, Lea and Blanchard, 1848; 8vo. pp. 670.

This, like every other scientific work, is to be regarded, critically, in a two fold point of view: first, as concerns the matter which it contains, and, second, as respects its arrangement, style, &c. In the latter point of view, it offers much room for criticism. The author, with the most praiseworthy intentions, has attempted to avoid the dullness, which, as he justly remarks, renders so many medical works tiresome and disgusting to the student. His effort has been, as he states in the introductory letter, to write with the same freedom and *abandon*, as if he were addressing familiarly any one of his pupils in his own library at home. As best adapted to such an object, he adopted an epistolary arrangement

That he has succeeded in writing a readable work, in our estimation, we have the best practical evidence in the fact, that, in spite of a deliberate resolve to postpone its perusal, having other more pressing matters on hand, we found ourselves taking it up day after day, at our leisure moments, until the volume was finished. If we may judge of others by our own experience, the student and practitioner will find it an attractive book. This consideration, while it disarms criticism of its acerbity, does not render it blind to literary blemishes. In the author's anxiety lest he should be tedious by confining himself too closely to naked scientific truths,

---

* ℞. Argenti Nitratis, gr. vj.

Extracti Conii, ʒj.

Aquæ Destillatæ, ℥i.

Misce, fiat injectio.

† ℞. Argenti Nitratis, gr. x.

Unguenti Cetacei, ℥ss.

Liquoris Plumbi, ʒss.

Misce, fiat unguentum.

he indulges in too many digressions and irrelevances. The work becomes thereby redundant and diffusive, and much too voluminous for the amount of instruction which it contains. The force of the latter is weakened by over dilution. Some of the dialogues which are introduced, strike us as in bad taste. We should pass this judgment upon the same conversations of physicians with patients, had they actually occurred within our hearing, but as fancy sketches for the imitation of pupils, they seem to us worse than superfluous. Let the readers of the work just imagine the members of the class, to whom the letters are dedicated, attempting to enact a scene like that in which "Miss Helen Blanque, at No. —, Chestnut street," is the patient!

The diction would have suited us far better had greater simplicity been observed. There is an appearance of scholastic pedantry which implies not a little pains in the choice of words, but considering the short period in which the work was written, much elaborateness of composition was impossible. The author must have written *currente calamo.* He must, therefore, have at his ready command philological stores, to an amount which is truly surprising, but here, as in other species of wealth, a constant profusion of expenditure is to be deprecated.

The style may be characterized as excessive. As the painters say, there is "too much warmpth of coloring" in it. This, the reader unacquainted with the fact that in this respect the author's writings are a true exponent of his mind, would be likely to attribute to affectation. But nothing is assumed. He writes as he lectures, and converses, to quote his own expression, "with his heart in his hand," and the earnestness and fervor which are impressed on the page, emanate from the heart. Not to extend this train of comment (the more, because we are far from making any pretensions as a literary critic,) we are free to say, that the character of the work is infinitely more pleasing to us than if written in a tame, spiritless manner, albeit, in the latter case, it were less obnoxious to criticism. We like to see the man in the book, and in the latter, as in the former, personal peculiarities, even amounting to eccentricities, sometimes are agreeable rather than offensive. The work before us, perhaps derives a piquancy from some of its faults. It shows the author to be a scholar, a man of genius, and entitled to an appellation which has conventionally a somewhat undefined, but nevertheless comprehensive and significant sense, and to which he evinces an especial partiality, viz, a gentleman. With this we leave the literary merits of the volume.

As regards matter, when it is considered that Prof. M. ranks among the most talented and learned of the profession of this country, and, we may safely add, of any country, and, moreover, that his experience in his particular province has been immense, it can hardly appear otherwise than that what he may write on the diseases of Females shall be deserving most respectful attention. But the same mental qualities which give rise to his peculiarities of style, render it necessary to make allowances for enthusiasm, and an imaginative tendency. We have surmised that some medical writers who possess the gift of racy, sparkling composition, are exposed to the danger of sometimes compromising that precision and correctness of expression so important in didactic instruction, for rhetorical point and ornament. This remark may possibly have some application in the present instance. But while we fear the work is not entitled to be considered as the best authority on all points, it is nevertheless replete with sound, practical precepts. During our perusal of the volume, we had occasion to mark several portions as containing questionable doctrine when compared with the experience and deductions of others. We leave them, however, to be particularized by others, as the present notice is not intended to embrace any reviewal of the work. Notwithstanding defects in style and matter, it is a work which will be read with pleasure and profit, and will confer honor on our national medical literature.

---

*On Bandaging, and other Operations of Minor Surgery*, by F. W. Sargent, M. D. Philadelphia: Lea & Blanchard, 1848.

The very best manual of Minor Surgery we have seen. An American volume, with nearly four hundred octavo pages of good, practical lessons, illustrated by about one hundred and thirty wood cuts. In these days of "trial," when a doctor's reputation hangs upon a clove hitch, or the roll of a bandage, it would be well, perhaps, to carry such a journal as Mr. Sargent's always in our coat-pocket, or, at all events, to listen attentively to his instructions at home.

The book consists of five principal divisions. The first comprising instruments used in dressing, surgical dressings, irrigation, disinfecting agents, &c., &c. The new disinfecting agent of M. Le Doyen, (a solution of the nitrate of lead) is especially recommended. The second division is devoted to bandages; the third to fracture apparatus; the fourth treats of dislocations and the means of reduction, and the fifth embraces all the remaining minor operations in surgery. The volume concludes with a brief chapter on the use of anæsthetic agents, in which we have only to complain of the injustice done to the memory of Dr. Wells, in not even alluding to his name in this connection. If Mr. Morton established the applicability of the vapour of ether as an anæsthetic agent, it is, we believe, no less certain that Dr. Wells established, *first*, the same fact in relation to the nitrous oxide gas, and it is to him, then, rather than to Mr. Morton, that the world is indebted for the discovery of the principle.—F. H. H.

